# Spatio-temporal distribution and influencing factors of norovirus outbreaks in Beijing, China from 2016 to 2020

**DOI:** 10.1186/s12879-023-08243-7

**Published:** 2023-05-02

**Authors:** Yanwei Chen, Baiwei Liu, Yu Wang, Yewu Zhang, Hanqiu Yan, Weihong Li, Lingyu Shen, Yi Tian, Lei Jia, Daitao Zhang, Peng Yang, Zhiyong Gao, Quanyi Wang

**Affiliations:** 1 Beijing Center for Disease Prevention and Control, No. 16 Hepingli Middle Street, Dongcheng District, Beijing, 100013 China; 2 Chinese Center for Disease Control and Prevention, Beijing, 102206 China

**Keywords:** Norovirus, Acute gastroenteritis, Outbreaks, Epidemiology, Geographical characteristic

## Abstract

**Background:**

Noroviruses are a leading cause of acute gastroenteritis (AGE) worldwide. The geographical characteristics of norovirus outbreaks in Beijing and their influencing factors remain unknown. This study aimed to explore the spatial distributions, geographical characteristics, and influencing factors of norovirus outbreaks in Beijing, China.

**Methods:**

Epidemiological data and specimens were collected through the AGE outbreak surveillance system in all 16 districts of Beijing. Data on spatial distribution, geographical characteristics, and influencing factors of norovirus outbreaks were analyzed using descriptive statistics methods. We measured spatial, geographical clustering of high- or low-value deviance from random distribution using *Z*-scores and *P*-values as statistical significance measures with Global Moran’s *I* statistics and Getis-Ord Gi in ArcGIS. Linear regression and correlation methods were used to explore influencing factors.

**Results:**

Between September 2016 and August 2020, 1,193 norovirus outbreaks were laboratory-confirmed. The number of outbreaks varied seasonally, typically peaking in spring (March to May) or winter (October to December). Outbreaks primarily occurred around central districts at the town level, and spatial autocorrelation was evident in both the entire study period and in individual years. Hotspots of norovirus outbreaks in Beijing were primarily found in contiguous areas between three central districts (Chaoyang, Haidian, Fengtai) and four suburban districts (Changping, Daxing, Fangshan, Tongzhou). The average population numbers, mean number of all schools, and mean number of kindergartens and primary schools for towns in central districts and hotspot areas were higher than those in suburban districts and non-hotspot areas respectively. Additionally, population numbers and densities of kindergartens and primary schools were influencing factors at the town level.

**Conclusions:**

Hotspots of norovirus outbreaks in Beijing were in contiguous areas between central and suburban districts with high populations, and high kindergarten and primary school densities were the likely driving forces. Outbreak surveillance needs to focus on contiguous areas between central and suburban districts with increased monitoring, medical resources, and health education.

## Background

Noroviruses are a leading cause of acute gastroenteritis (AGE) worldwide, accounting for a large proportion of sporadic AGE cases and outbreaks in recent years [1]. Approximately 179 million AGE episodes occur annually in the United States, and norovirus, the leading cause of single-etiology outbreaks, is responsible for 68% of these episodes [2]. In Europe, approximately 15 million foodborne illnesses occur each year, with the most frequent cause being diarrhea induced by norovirus [3].

A meta-analysis indicated that the prevalence of norovirus in cases of AGE in developing countries was 17% [4]. Additionally, in lower-middle-income countries, norovirus was detected in 15% of cases and 8% of healthy controls, while 11% of symptomatic cases and 9% of asymptomatic controls were norovirus positive in low-income countries [5]. In China, the incidence rate of norovirus was six cases per 100 person-years in the general population and 16 cases per 100 person-years in children aged < 5 years [6].

In 2011, a hospital-based surveillance network was established in all 16 districts of Beijing, China for monitoring sporadic AGE cases caused by norovirus and rotavirus [6]. Moreover, the Beijing Center for Disease Control and Prevention (CDC) and 16 district CDCs developed an AGE outbreak surveillance network in April 2014 [7]. From this network, data showed that norovirus accounted for 86.7% of laboratory-confirmed AGE outbreaks from 2014 to 2017 in Beijing [7].

Norovirus outbreaks show seasonality, with peaks during winter months [8]. The virus is highly contagious and can be transmitted via the fecal–oral route, person-to-person, or through contaminated water, food, or surfaces [9]. The main symptoms of norovirus infections are vomiting and diarrhea, which are usually self-limiting and of 1 to 3 days’ duration [10]. However, older populations, immunocompromised individuals, and children younger than 5 years may suffer from severe or prolonged illness [11]. Outbreaks caused by norovirus are often reported in schools, kindergartens, hospitals, restaurants, and daily care centers [6].

Our previous study showed that outbreaks are usually reported in the winter and spring, with 88.89% of outbreaks reported in kindergartens and schools [12]. The risk of outbreaks in suburbs and outer suburbs were 1.84 times and 3.78 times as high as those in urban areas, respectively [12]. However, the geographical characteristics of norovirus outbreaks in Beijing and their influencing factors have not yet been analyzed.

In recent decades, geographic information systems (GIS) and spatiotemporal techniques have been widely used in the surveillance and investigation of infectious diseases [13–15]. These technologies may help us understand epidemiological characteristics and explore hotspots of outbreaks of infectious diseases [16]. Norovirus outbreaks are spatially patterned, and these patterns are associated with specific environmental factors [17]. We conducted this study based on norovirus surveillance data collected between September 2016 and August 2020 in Beijing to better understand the spatial characteristics and influencing factors of norovirus outbreaks.

## Methods

### AGE outbreak surveillance

AGE outbreak surveillance was established in Beijing in April 2014, and district-level Centers for Disease Control and Prevention finished the preliminary epidemiological investigation and detection of fecal specimens. Patients with diarrhea, defined as three or more loose stools within a 24 h period, and/or vomiting, defined as one or more episodes, were designated as AGE cases. An AGE outbreak was defined as the occurrence of three or more epidemiologically linked cases of AGE within a 3-day period. A norovirus outbreak was confirmed if more than two AGE cases tested positive for the norovirus. Norovirus was tested by real-time reverse transcription polymerase chain reaction (PCR).

In this study, a norovirus outbreak surveillance year was defined as starting on September 1 and ending on August 31 of the following year. Data on norovirus outbreaks reported between September 2016 and August 2020 were collected and imported into the WPS Spreadsheets 2016 (Kingsoft Inc., Beijing, China) for manipulation and graphing. Additionally, the annual incidence of norovirus outbreaks per 100,000 people in each town was calculated.

### Statistical analyses

Statistical analyses were performed using the SPSS v20.0 software (SPSS Inc., Chicago, IL, USA). Statistical significance was assessed using an α level of 0.05 for all analyses and set at *P* < 0.05 (two-tailed). The distribution of data was tested using one-sample Kolmogorov–Smirnov (KS) test. Means for different groups were compared using *t*-tests.

### Map of Beijing

The map of Beijing at the town level was purchased and used with permission from the Beijing Institute of Surveying and Mapping. Beijing has geographical coordinates of N39°56' and E116°20', is located in a warm temperate zone, and has a semi-humid and semi-arid monsoon climate [18]. There are 16 districts in Beijing. including six central and 10 suburban districts. Dongcheng, Xicheng, Chaoyang, Haidian, Fengtai, and Shijingshan districts are central districts, and Mentougou, Fangshan, Tongzhou, Shunyi, Changping, Daxing, Huairou, Pinggu, Miyun, and Yanqing districts are suburban districts [19].

### Population numbers and schools in Beijing

Data on the 0–14 age group at the district level and the populations of all age groups at the town level were acquired from the open database of the seventh national census, which was held in November 2020, and from the official websites of the 16 districts of the People’s Government of Beijing Municipality. The number of total schools, kindergartens, and primary schools in each town were similarly collected from the official page of the People’s Government of Beijing Municipality.

### Spatial analysis of norovirus outbreaks

Analyses of the spatial distribution of norovirus outbreaks were conducted using ArcGIS [20] software (version 10.6; ESRI, Redlands, CA, USA), based on a town-level polygon map.

### Conceptualization of specific spatial relationships

The spatial weight matrix file was generated using spatial statistics tools in the ArcGIS toolbox. The K-nearest neighbor method was used to define the conceptualization of spatial relationships. The distance between each element and its neighbors was calculated using the Euclidean distance formula.

### Moran's *I* statistic

To explore the spatial characteristics of annual norovirus outbreaks, we used spatial autocorrelation to examine whether the distribution pattern was clustered, dispersed, or random. Moran’s *I* index values were calculated and a matrix file was used to define the conceptualization of spatial relationships. When *p*-values were statistical significant, positive Moran’s *I* index values indicated a cluster tendency. Otherwise, index values indicated a dispersed tendency.

### Hotspot analysis (Getis-Ord Gi*)

The spatial hotspots and cold spots of outbreaks were identified, considering the weighted features of the matrix file, using *Z*-scores and *P*-values as statistical significance measures with Getis-Ord Gi in ArcGIS. High *Z*-scores and a *P* < 0.05 indicated hotspots with statistical significance. In this study, we focused on hotspots.

### Influencing factors analysis

We analyzed the association between the annual average number of norovirus outbreaks and variables (population, density of population, numbers of schools, density of schools) one by one using simple linear regression analysis. Variables that showed significance (*P* < 0.05) in the univariate analysis were included for stepwise multiple linear regression analysis, collinearity statistics was conducted simultaneously, and variables with a variance inflation factor (VIF) above 10 were excluded from the multiple linear regression analysis. Pearson and Spearman correlation tests were used to analyze the relationship between outbreaks and population and population density of the 0–14 age group.

The area (km^2^) of districts and towns was calculated using the calculation geometry tool in ArcGIS. Population density was defined as the population number divided by area of the town or district, and the density of schools was defined as the number of schools divided by area of the town. Number of schools per population was defined as the number of schools divided by the population (100,000) of the town.

## Results

### Norovirus outbreaks in Beijing from 2016 to 2020

Between September 2016 and August 2020, 1,954 AGE outbreaks were reported. Of these, 1,193 (61.05%) were laboratory-confirmed as norovirus and 16 (0.82%) were mixed infections with norovirus and other pathogens. In our study, we focused on the 1193 laboratory-confirmed norovirus outbreaks, which showed seasonal peaks in spring (March to May, 554 outbreaks, 46.44%) and winter (October to December, 390 outbreaks, 32.69%). Of the 1,193 norovirus outbreaks, 580 (48.62%) occurred in kindergartens, followed by 449 (37.64%) in primary schools, 76 (6.37%) in middle schools, 19 (1.59%) in comprehensive schools, 14 (1.17%) in universities, and five (0.42%) in vocational schools. The remaining 50 (4.19%) outbreaks occurred at sites such as hospitals, nursing homes, and companies. A total of 726 (60.85%) outbreaks occurred in six central districts and 467 (39.15%) outbreaks occurred in 10 suburban districts.

### Spatial distributions of norovirus outbreaks at the town level in Beijing

**Fig. 1:**
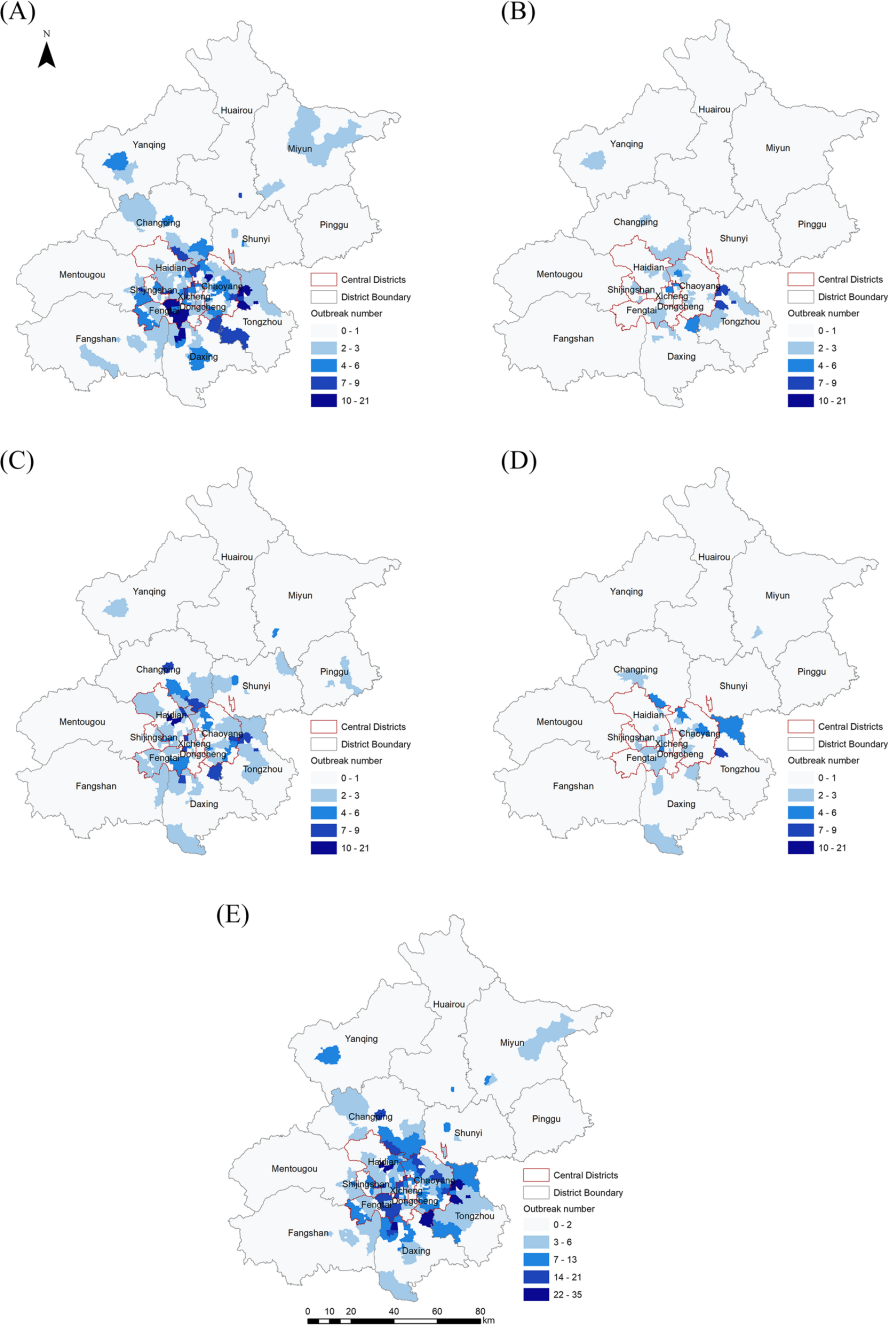
Distribution of norovirus outbreaks at the town level in Beijing, from 2016 to 2020 (**A**), (**B**), (**C**), and (**D**) show the distribution of norovirus outbreaks from 2016 to 2017, 2017 to 2018, 2018 to 2019, and 2019 to 2020, respectively. (**E**) shows the distribution of the total reported norovirus outbreaks from 2016 to 2020

According to the distribution of the total reported norovirus outbreaks from 2016 to 2020, outbreaks primarily occurred around the central districts, with the same pattern evident for every year of surveillance (Fig. 1).

### Global Moran’s *I* statistic

The spatial distributions of norovirus outbreaks at the town level in Beijing indicated spatial autocorrelation characteristics for both the entire study period and individual years. *Z*-scores and *P*-values generated by global Moran's *I* statistics for each surveillance year and the entire study period were statistically significant (Table 1).

**Table 1:** Global Moran’s *I* statistics of norovirus outbreaks at the town level in Beijing from 2016 to 2020

Years	Moran’s *I*	Variance (*S*^2^)	*Z*	*P*
2016–2017	0.202	0.001	8.002	< 0.001
2017–2018	0.154	0.001	6.178	< 0.001
2018–2019	0.148	0.001	6.108	< 0.001
2019–2020	0.090	0.001	3.671	< 0.001
Total	0.265	0.0012	10.412	< 0.001

### Hotspot analysis (Getis–Ord Gi*)

**Fig. 2:**
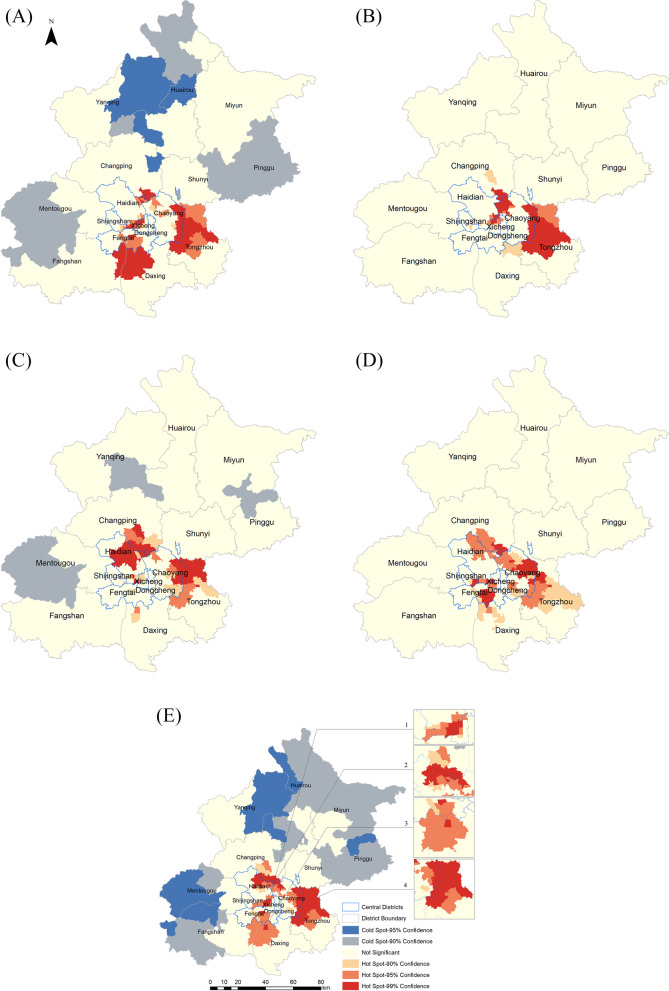
Hotspots of norovirus outbreaks on town level in Beijing, from 2016 to 2020 (**A**), (**B**), (**C**), and (**D**) show hotspots of norovirus outbreaks from 2016 to 2017, 2017 to 2018, 2018 to 2019, and 2019 to 2020, respectively. (**E**) shows hotspots for the total reported norovirus outbreaks from 2016 to 2020

From 2016 to 2017, 42 towns, primarily distributed in contiguous areas of different districts, were recognized as hotspots. These included seven towns in the contiguous areas of Haidian and Xicheng and three towns in the contiguous areas of Haidian and Shijingshan. Others were found in contiguous areas of the following: Haidian, Chaoyang, and Changping districts (five towns); Chaoyang and Tongzhou districts and towns in the north of Tongzhou (14 towns); Fengtai, Fangshan, and Daxing districts and towns in the northwest of Daxing (12 towns); and one town in the central area of Chaoyang district (Fig. 2).

From 2017 to 2018, 31 hotspots were observed, comparatively lower than from 2016 to 2017. Hotspots were primarily found in contiguous areas of Xicheng and Haidian district (six towns), Dongcheng and Xicheng district (three towns), Chaoyang and Changping districts (three towns), and five towns in the northwestern parts of Chaoyang district. Additionally, hotspots were found in contiguous areas of the Chaoyang and Tongzhou districts (nine towns) and in the northern parts of Tongzhou (five towns) (Fig. 2).

Moreover, from 2018 to 2019, 33 towns were found to be hotspots and were primarily distributed in the north and east of the central districts. The number of hotspots in or close to Haidian (17 towns) increased significantly compared with the previous year, distributed in central areas of Haidian district (nine towns) and on contiguous areas of Haidian and Changping (six towns) and Haidian and Xicheng district (two towns). In the contiguous areas of the Chaoyang and Tongzhou districts, 10 towns were hotspots. One town was a hotspot in the contiguous areas of Chaoyang and Changping. We also found hotspots in the north of Tongzhou (three towns), Daxing (one town), and in the middle of Changping (one town) (Fig. 2).

From 2019 to 2020, 34 towns in the Haidian, Changping, Chaoyang, and Tongzhou districts were deemed hotspots. In the contiguous areas of Shijingshan, Xicheng, and Fengtai districts, the distribution of hotspots was ring-like and included 14 towns. Additionally, one town in the north of Daxing was a hotspot (Fig. 2).

We analyzed the accumulated data of the outbreaks from 2016 to 2020. The results showed that 58 hotspots were primarily found in contiguous areas between three central districts (Chaoyang, Haidian, Fengtai) and four suburban districts (Changping, Daxing, Fangshan, Tongzhou), which we separated into four parts. The first part was in the central districts of Beijing, in the contiguous areas of Xicheng, Shijingshan, and Haidian districts, of which 14 towns were hotspots. Second, to the north of the central districts, 14 towns were hotspots in the contiguous areas of the Haidian, Changping, and Chaoyang districts. Third, in southeastern Beijing, on the boundary areas of the Fengtai, Daxing, and Fangshan districts, 11 towns were hotspots. Finally, in the contiguous areas of the Chaoyang and Tongzhou districts, and together for the northern parts of Tongzhou, 19 towns were hotspots. We considered these 58 towns as hotspot areas in subsequent analyses (Fig. 2).

### Population distribution in Beijing

**Fig. 3:**
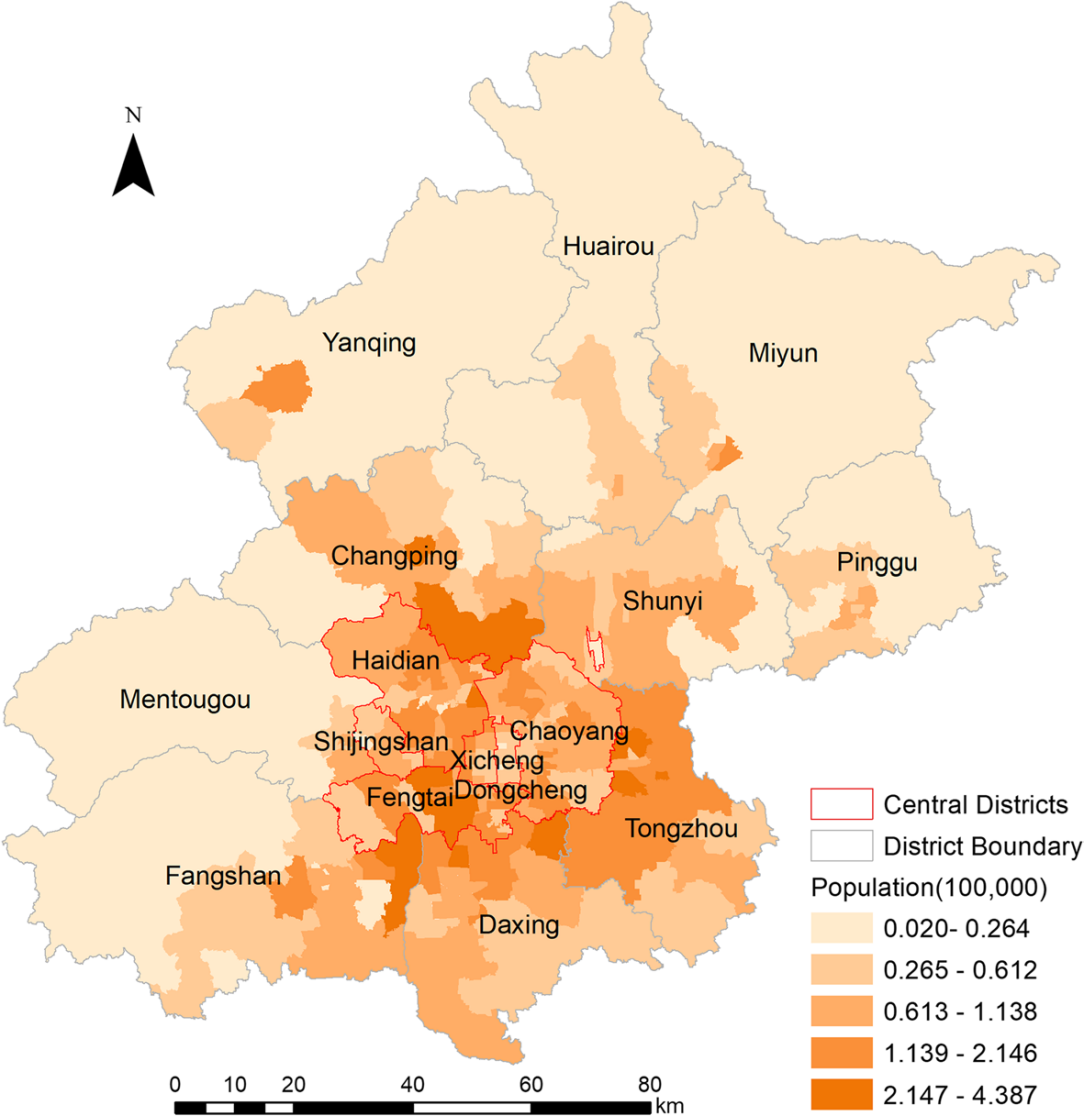
Distribution of population at the town level in Beijing: data from the seventh national census

The population distribution in Beijing is shown in Fig. 3. The average population for towns in central districts was 0.085 million, which was higher than that in suburban districts (0.059 million) (*t* = 3.43, *P* = 0.001). Compared with the average population in non-hotspot areas (0.055 million), the population in hotspots was considerably higher (0.131 million, *t* = 6.54, *P* < 0.001).

### Distribution of schools in Beijing

**Fig. 4:**
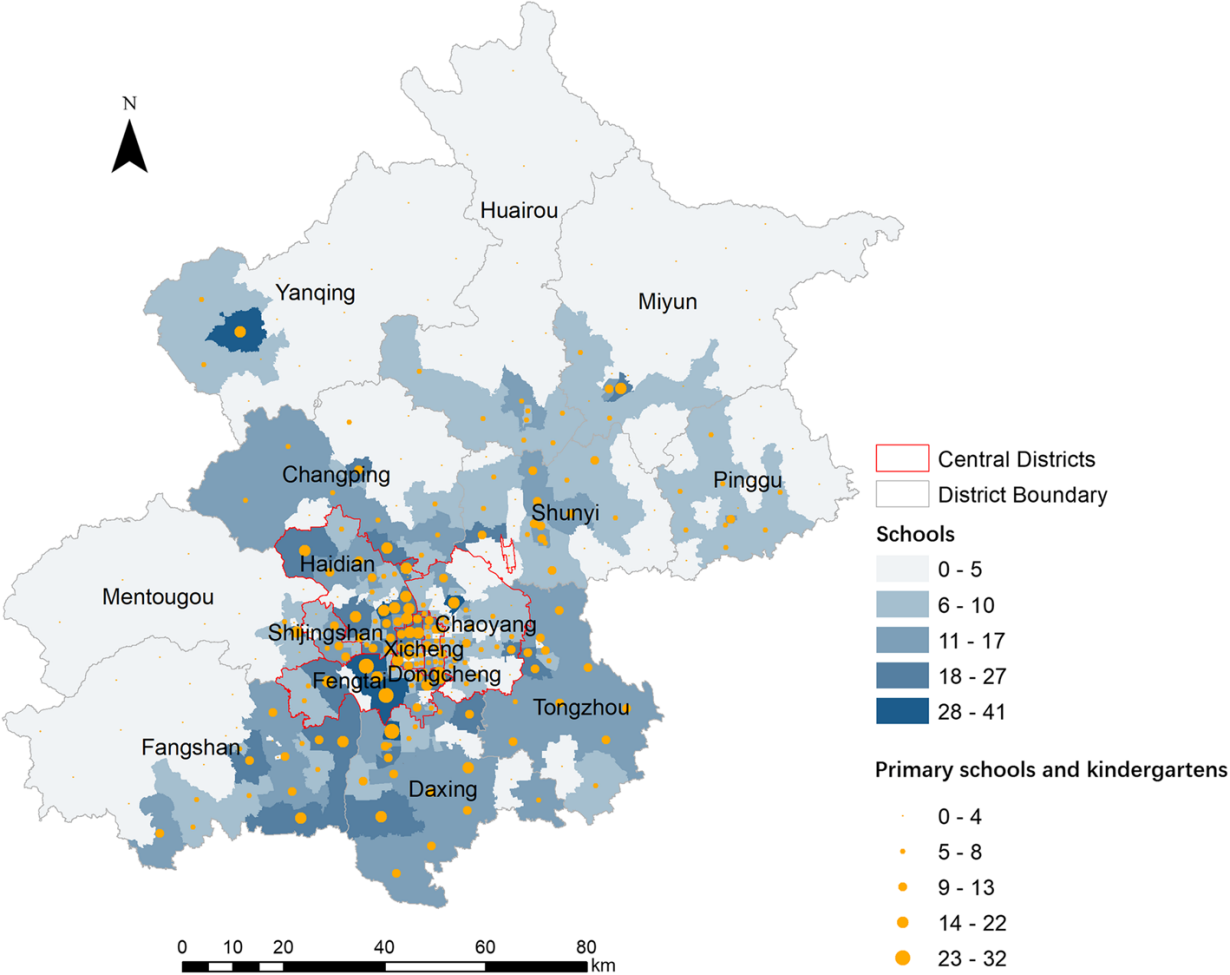
Distribution of schools at the town level in Beijing

We calculated the number of schools, kindergartens, and primary schools in each town. The mean number of total schools, mean number of kindergartens and primary schools in central district towns and hotspot areas were higher than those in suburban districts and non-hotspot areas respectively. Density of all schools (/km^2^), density of kindergartens and primary schools (/km^2^) in central district towns were higher than those in suburban districts. However, number of kindergartens and primary schools per population (/100,000) in central district towns and hotspot areas were lower than those in suburban districts and non-hotspot areas respectively (Table 2). The distribution of schools on town level in Beijing is shown in Fig. 4.

**Table 2:** Comparation of schools and populations in different regions of Beijing

Variables	Suburban district towns	Central district towns	Non-hotspot towns	Hotspot towns
N of towns	186	130	258	58
Number of all schools				
sum	1481	1524	2206	799
mean	7.96	11.72	8.55	13.78
*t*	4.61		5.31	
*P*	< 0.001		< 0.001	
Number of kindergartens and primary schools				
sum	1080	1019	1554	545
mean	5.81	7.84	6.02	9.4
*t*	3.44		4.81	
*P*	0.001		< 0.001	
Number of kindergartens and primary schools per population (/100,000)				
mean	19.15	11.06	17.53	8.25
*t*	5.42		7.92	
*P*	< 0.001		< 0.001	
Number of all schools per population (/100,000)				
mean	24.37	16.76	23.25	12.31
*t*	4.11		7.48	
*P*	< 0.001		< 0.001	
Densities of kindergartens and primary schools (/km^2^)				
mean	0.28	1.62	0.77	1.07
*t*	-10.00		-1.68	
*P*	< 0.001		0.094	
Densities of all schools (/km^2^)				
mean	0.40	2.46	1.17	1.61
*t*	-10.62		-1.69	
*P*	< 0.001		0.092	
Population (100,000)				
sum	109.05	109.89	142.97	75.96
mean	0.59	0.85	0.55	1.31
*t*	3.43		6.54	
*P*	0.001		< 0.001

### Influencing factors analysis

The influence of each variable was statistically significant in simple linear regression analysis (Table 3). However, the multiple linear regression analysis showed that population (100,000) (*t* = 17.121, *P* < 0.001, 95%CI 1.192–1.502) and densities of kindergartens and primary schools (/km^2^) (*t* = 3.988, *P* < 0.001, 95%CI 0.088–0.258) were influencing factors for norovirus outbreaks on town level. In contrast, density of population (10,000/km^2^) (*t* = 0.018, *P* = 0.985), numbers of kindergartens and primary schools (*t* = -0.802, *P* = 0.423), total number of schools (*t* = 1.141, *P* = 0.255), number of kindergartens and primary schools per population (/100,000) (*t* = 0.234, *P* = 0.815), number of all schools per population (/100,000) (*t* = 0.254, *P* = 0.799) showed no statistically significant difference. The VIF for densities of all schools (/km^2^) was 18.907, indicating high collinearity, and this variable was excluded from multiple linear regression analysis. We conducted stepwise regression analysis again without the densities of all schools (/km^2^), and the coefficients for variables remained unchanged.

**Table 3:** Linear regression analyses on influencing factors of norovirus outbreaks at the town level in Beijing from 2016 to 2020

**Variables**	simple linear regression								multiple linear regression				
	***β***	***S***_**‾x**_	***t***	***P***_**1**_	**95%CI**	***R***^**2**^	***F***	***P***_**2**_	***β***	***t***	***P***	**Collinearity Statistics**	
												**Tolerance**	**VIF**
Densities of kindergartens and primary schools (/km^2^)	0.258	0.06	4.315	< 0.001	0.557–0.903	0.056	18.617	< 0.001	0.158	3.988	< 0.001	0.987	1.013
Population (100,000)	0.383	0.08	17.291	< 0.001	1.226–1.540	0.488	289.980	< 0.001	0.680	17.121	< 0.001	0.987	1.013
Densities of all schools (/km^2^)	0.168	0.041	4.134	< 0.001	0.088–0.248	0.052	17.089	< 0.001	-0.114	-0.661	0.509	0.053	18.907
Density of population (10,000/km^2^)	0.478	0.073	6.530	< 0.001	0.334–0.622	0.120	42.641	< 0.001	0.001	0.018	0.985	0.328	3.048
Number of kindergartens and primary schools	0.135	0.013	10.427	< 0.001	0.109–0.160	0.257	108.718	< 0.001	0.044	0.802	0.423	0.512	1.954
Number of all schools	0.104	0.009	11.663	< 0.001	0.086–0.121	0.302	136.034	< 0.001	0.068	1.141	0.255	0.436	2.292
Number of kindergartens and primary schools per population (/100,000)	-0.027	0.005	-5.604	< 0.001	-0.037– -0.018	0.091	31.400	< 0.001	0.010	0.234	0.815	0.805	1.242
Number of all schools per population (/100,000)	-0.023	0.004	-5.598	< 0.001	-0.032– -0.015	0.091	31.334	< 0.001	0.011	0.254	0.799	0.792	1.263

*VIF* Variance inflation factor

^*^P_1_ and *P*_2_ represents the *P*-value for significance of variables and the univariate regression model respectively

One-sample KS tests showed that the 0–14 age group (*t* = 0.176, *P* = 0.198) and outbreak number (*t* = 0.212, *P* = 0.053) data for 16 districts were normally distributed, and the population density data of the 0–14 age group (*t* = 0.282, *P* = 0.001) was abnormally distributed. There were correlations between outbreak number and the 0–14 age group population (*r* = 0.910, *P* < 0.001) and the 0–14 age group population density (*r* = 0.821, *P* < 0.001), as demonstrated by Pearson and Spearman correlation tests, respectively (Table 4).

**Table 4:** Population and population density of the 0–14 age group and norovirus outbreaks in Beijing from 2016 to 2020

District	Outbreaks	Population	Population density (/km^2^)
Dongcheng	48	98,290	2347.381
Xicheng	126	157,912	3124.291
Chaoyang	261	395,192	851.336
Fengtai	85	219,680	719.043
Shijingshan	37	64,509	765.664
Haidian	169	371,111	862.315
Mentougou	22	44,760	30.930
Fangshan	30	169,217	84.881
Tongzhou	121	222,726	246.079
Shunyi	41	155,318	153.899
Changping	81	235,965	175.903
Daxing	109	236,916	228.884
Huairou	11	51,932	24.495
Pinggu	10	60,760	64.124
Miyun	27	66,747	30.009
Yanqing	15	40,472	20.297
Total	1193	2,591,507	2347.381

## Discussion

Globally, norovirus-associated AGE outbreaks occur periodically [1]. From 2009–2017, norovirus was the suspected or confirmed etiology of 47% of AGE outbreaks in the United States [21]. Norovirus outbreaks have become an important public health concern in China. In China, from January 2014 to December 2017, norovirus was associated with 89.02% (616/692) of AGE outbreaks reported to the National Public Health Emergency Event Surveillance System (PHEESS) [6], and from October 2016 to September 2018, 556 norovirus outbreaks were reported [22]. During our study period, we found that norovirus caused a large proportion of AGE outbreaks in Beijing, ranging from 28.11%–89.30%, with an average of 61.05%. This finding is consistent with epidemic trends reported in other parts of China [23].

Norovirus outbreaks typically occur in crowded settings where many people gather. In Japan and South Korea [24, 25], similar to Beijing and various Chinese provinces [22, 26], outbreaks generally occur in childcare centers and schools. However, in developed countries, norovirus outbreaks mainly occur in healthcare facilities, [2, 11, 27, 28], which may reflect the differences in the sensitivity and coverage among surveillance systems. A specific norovirus surveillance system has not been established for healthcare facilities in China [26]. At present, the care home structure in China is also very different with that in developed countries, and most elderly people live at home with family members instead of in healthcare centers. With the exception of winter and summer vacations, students often gather in classrooms, making them more susceptible to noroviruses. Therefore, implementing prevention and control measures during school semesters is critical [29]. In addition, in early 2016, the Technical Guidelines for Norovirus Infection Outbreak Investigation and Prevention and Control were issued in China [9]. Since then, the number of outbreaks in schools and kindergartens has increased, which may be related to the variation of virus strains as well as increased outbreak investigations [29].

Furthermore, rainfall, relative humidity, and changes in temperature are important factors in norovirus seasonality [8, 30, 31]. Norovirus outbreaks in Beijing tend to peak in the spring and winter, which corresponds with other provinces of China [32, 33], but differs with Europe and Canada [27, 34], where outbreaks tended to peak in the winter. In addition to the influence of meteorological factors, crowding of susceptible populations, and long-term indoor activities, such as in kindergartens and primary schools, have caused increased human-to-human transmission [8].

The spatial distribution of hotspots of norovirus outbreaks varied geographically across Beijing and were mainly concentrated in the junctions of central districts and suburban districts, which were densely populated and relatively lacking in community health services [35]. A similar situation was observed in Tokyo, where a higher number of norovirus cases were reported in peripheral areas that surrounded the most populated central area [36]. In addition, the distribution of hotspots of hand, foot, and mouth disease (HFMD) in Beijing was similar to that of norovirus outbreaks in our study [37]. The population density and proportion of the student population were also factors influencing HFMD in mainland China [38]. In general, high population densities and the relative lack of community health services may be conducive to norovirus outbreaks as well as other infectious diseases. Beijing is a large city with 21.89 million inhabitants, and our findings may provide a reference for similar cities in the prevention and control of infectious diseases. Attention to these areas is vital when formulating monitoring plans, allocating medical resources, and conducting health education.

Our study showed that population and densities of kindergartens and primary schools (/km^2^) were influencing factors in norovirus outbreaks. There were strong correlations between outbreak numbers and the 0–14 age group population and the 0–14 age group population density. These results suggest that kindergarten children and primary school students play an important role in norovirus outbreaks. These children stay in crowded, enclosed classrooms where viruses, such as norovirus, are easily transmitted by person-to-person contact, causing outbreaks [29]. In addition, a high number of kindergartens and primary schools may also accelerate the spread of norovirus. Many students often participate in various extracurricular training classes in Beijing, which are also crowded, enclosed, and a common outbreak setting. These students typically arrive from different schools, which may promote the spread between schools. Studies have demonstrated that thorough disinfection of surfaces and enhanced hand hygiene can reduce the possibility of viral transmission [39]. At the same time, the monitoring of symptoms in schools and kindergartens may play an important role in the early detection of outbreaks.

As norovirus is mainly transmitted person-to-person, high population numbers increase the opportunities for transmission. In other provinces of China, a significantly higher number of norovirus outbreaks occurred in southern and eastern China, predominantly in Guangdong and Zhejiang provinces [40]. Additionally, higher economic development results in larger populations, which may accelerate the spread of norovirus.

### Limitations

Our study had several limitations. First, the map we used contained the administrative region in Beijing in 2012, and several townships were divided in later years, which may possibly decrease or increase the risk level in some towns. Second, there may have been differences in the awareness of epidemic reporting and related management in different towns and districts, which may have influenced the number of reported norovirus outbreaks. Third, our study found that higher population numbers increased the risk of transmission; however, population density was not found to be an influencing factor. As population density was calculated according to the permanent population and areas of towns and the floating population were not included, population density may have been underestimated. Finally, data on age group populations at the town level are not publicly available, and only data on the 0–14, 15–59 and ≥ 60 age group populations at the district level are available.

## Conclusions

Norovirus outbreaks in Beijing showed spatial autocorrelation characteristics, and hotspots were in contiguous areas between central and suburban districts with high populations, and high kindergarten and primary school densities were the likely driving forces. Outbreak surveillance needs to focus on contiguous areas between central and suburban districts with increased monitoring, medical resources, and health education.

## Data Availability

The datasets used and/or analyzed during the current study are available from the corresponding author on reasonable request.

## Abbreviations

AGE: Acute gastroenteritis
CDC: Center for disease control and prevention
GIS: Geographic information systems
PCR: Polymerase chain reaction
VIF: Variance inflation factor
HFMD: Hand, foot, and mouth disease
